# Monitoring Distribution of the Therapeutic Agent Dimethyl Sulfoxide via Solvatochromic Shift of Albumin-Bound Indocyanine Green

**DOI:** 10.3390/s23187728

**Published:** 2023-09-07

**Authors:** Jaedu Cho, Farouk Nouizi, Chang-Seok Kim, Gultekin Gulsen

**Affiliations:** 1 Tu and Yuen Center for Functional Onco-Imaging, Department of Radiological Sciences, University of California, Irvine, CA 92697, USAfnouizi@hs.uci.edu (F.N.); 2 Department of Cogno-Mechatronics Engineering, Pusan National University, Busan 607-735, Republic of Korea; ckim@pusan.ac.kr

**Keywords:** fluorescence imaging, multispectral and hyperspectral imaging, solvatochromic shift, tunable lasers, spectroscopy, DMSO sensing, drug uptake monitoring

## Abstract

We recently developed a novel hyperspectral excitation-resolved near-infrared fluorescence imaging system (HER-NIRF) based on a continuous-wave wavelength-swept laser. In this study, this technique is applied to measure the distribution of the therapeutic agent dimethyl sulfoxide (DMSO) by utilizing solvatochromic shift in the spectral profile of albumin-bound Indocyanine green (ICG). Using wide-field imaging in turbid media, complex dynamics of albumin-bound ICG are measured in mixtures of dimethyl sulfoxide (DMSO) and water. Phantom experiments are conducted to evaluate the performance of the HER-NIRF system. The results show that the distribution of DMSO can be visualized in the wide-field reflection geometry. One of the main purposes of the DMSO is to act as a carrier for other drugs, enhancing their effects by facilitating skin penetration. Understanding the solubility and permeability of drugs in vivo is very important in drug discovery and development. Hence, this HER-NIRF technique has great potential to advance the utilization of the therapeutic agent DMSO by mapping its distribution via the solvatochromic shift of ICG. By customizing the operational wavelength range, this system can be applied to any other fluorophores in the near-infrared region and utilized for a wide variety of drug delivery studies.

## 1. Introduction

Dimethyl sulfoxide (DMSO) is one of the most commonly used pharmaceutical drugs in life sciences. It has a wide spectrum of pharmacological effects, including anti-inflammatory effects, local analgesia, weak bacteriostasis, and, most importantly, membrane penetration. DMSO is used in animals and humans through various routes, including dermal and oral, and it is also utilized to enhance the absorption of many other chemicals along these same routes. At low concentrations, DMSO exhibits anti-inflammatory, analgesic, diuretic, vasodilator, anti-platelet aggregation, radio-protective, and muscle-relaxing properties [1]. The observed beneficial effects on skin rejuvenation and recovery from thermal injury might be explained by its effective property of being a vigorous scavenger of hydroxyl free radicals. Accordingly, it has been applied for a variety of purposes, such as in the treatment of musculoskeletal and dermatological diseases, cryopreservation of stem cells, treatment of interstitial cystitis, treatment of increased intracranial pressure, and many more [2,3,4,5,6,7].

One of the principal uses of DMSO is as a vehicle for other drugs, thereby enhancing the effect of the drug and aiding the penetration of other drugs into the skin. For example, it is now well established as a penetration enhancer in topical pharmaceutical formulations. The permeability of drugs to the skin essentially requires that the retained epidermis be diffused by the drug. DMSO is a safe and effective mechanism for facilitating the transdermal delivery of both hydrophilic and lipophilic medications to provide localized drug delivery [8]. The effect on cell membranes depends on the concentration of DMSO, ranging from an increased bilayer fluidity at lower concentrations to the formation of water pores and the extraction of lipid molecules at higher concentrations [9]. Therefore, the capability of measuring the local concentration of DMSO in vivo is important. In addition, DMSO is also utilized in other delivery strategies to overcome issues of solubility such as particle size reduction by micronizing the active compound or the use of nanocarriers as a vehicle. As one of those methods, the supercritical antisolvent (SAS) technique utilizes DSMO regularly [10]. Another example of such a technique is SALT, solvent-assisted active loading technology, which was developed to promote active loading of poorly soluble drugs in the liposomal core to improve the encapsulation efficiency and formulation stability [11].

Depending on the application type and its dose, DMSO can induce adverse effects, including the generation of oxidative stress and cytotoxicity [12]. When used for the preservation of liquid-nitrogen-frozen stem cells, for example, DMSO was associated with toxicity in the transplant recipient in a prospective noninterventional study in 64 European Blood and Marrow Transplant Group centers undertaking autologous transplantation on patients with myeloma and lymphoma [13]. Meanwhile, Madsen et al. reviewed a total of 109 different clinical studies involving intravenous, transdermal, oral, and intravenous administration of DSMO. They showed that gastrointestinal and skin reactions were the most commonly reported adverse reactions [14]. Most importantly, their investigation confirmed a relationship between the given dose of DMSO and the occurrence of adverse reactions.

Since the dose plays an important role in the occurrence of adverse reactions, it is important to have a technique that can provide the distribution and dose of DSMO. Moreover, DSMO dose has a critical effect on cell membranes and hence the delivery of the drugs. The solubility of DMSO-mixed water-insoluble drugs depends on the local residual level of DMSO, and the permeability of the drugs decreases with the precipitation of the drugs in biological media. Therefore, a technique that can monitor the DSMO concentration will play a critical role in the investigation of the optimum dose of DMSO for a particular application by increasing the efficiency of drug delivery and reducing adverse reactions. Such a technique may also guide the applied DMSO dose in real time during therapeutic applications. 

Saar et al. have demonstrated molecular imaging visualizing DMSO with a stimulated Raman scattering technique [3]. However, this nonlinear microscopy is inadequate for wide-field imaging with an organ-level field of view. It has long been known that UV/vis/near-IR absorption spectra of chemical compounds may be influenced by the surrounding medium and that solvents can bring about a change in the position, intensity, and shape of absorption bands [15]. Measuring the environment polarity in different media has been studied extensively with optical fluorescent dyes using solvatochromism [4]. Indeed, optical dyes are used for this purpose since their molecular spectral profiles are significantly affected by the local polarity of biological media. These compounds whose absorption and emission spectra depend on the environmental polarity are known as solvatochromic dyes, which can operate in a wide range of the optical spectrum, including ultraviolet, visible, and near-infrared (NIR, ~650–950 nm). However, it is challenging to perform solvatochromic-dye-mediated polarity measurements in vivo since these dyes unfortunately have heavily overlapping excitation and emission spectra and exhibit weak solvatochromic shifts in their spectral profiles. Moreover, tissue optical properties affect the measured fluorescence spectra [5,6]. 

Establishing a regular solvatochromic response to various DSMO concentrations can serve as a technique for DMSO sensing. Truksa et al. recently investigated the change in absorption and emission spectra of lumazine, alloxazine, and their cyanated or fluorinated derivatives with DSMO [16]. They measured the modulated spectroscopic properties by varying the water concentration in DMSO solutions. Cyano-substituted lumazine has shown a remarkable sensitivity for the relative DMSO–water concentrations, including an unexpected behavior in the solution containing 65% water and 35% DMSO. It seemed like water–DMSO trimers were formed at this particular concentration and this sensitivity was the manifestation of changes in the local structure of the solvent, i.e., the formation or dissolution of H_2_O clusters. The authors concluded that together with its enhanced water solubility, the cyanated lumazine derivate could be used for non-destructive DMSO detection in vitro for applications [16]. 

In this work, our aim is to develop an in vivo DSMO sensing technique utilizing the solvatochromic response of fluorescence dyes. Among the various solvatochromic dyes, Indocyanine green (ICG) is the most widely used NIR dye for fluorescence imaging [17,18,19,20,21,22,23]. Its absorption peak is around 800 nm, where biological tissue attenuates the fluorescence less and generates low autofluorescence [8]. Furthermore, hemoglobin absorption stays low and has an isosbestic point at 800 nm. Despite these spectroscopic merits of ICG in the 800 nm spectral window, the use of ICG has been limited to visualize blood perfusion volume and tissue-permeability-rate-based physiological assessment due to the lack of molecular specificity. 

To be able to measure this complex solvatochromic response of ICG, a fluorescence lifetime imaging technique has been introduced [24]. However, this fluorescence lifetime technique requires either a pulsed laser or a radio frequency amplitude-modulated light source. In addition, expensive high-speed electronics are necessary to measure the time domain or frequency domain signals. We previously developed a novel hyperspectral excitation-resolved near-infrared fluorescence imaging system (HER-NIRF) based on a continuous-wave wavelength-swept laser [25]. Based on a semiconductor optical amplifier and a wavelength selector consisting of a holographic transmission grating, a reflection mirror, and a Galvo-scanner mirror, this laser source can provide any wavelength between 784 and 805 nm, a range that is particularly optimal for the ICG absorption peak. In this study, this technique is applied to measure the distribution of the therapeutic agent dimethyl sulfoxide (DMSO) by utilizing the solvatochromic shift in the spectral profile of ICG. Since ICG is a U.S. Food and Drug Administration-approved fluorescent dye, the technique presented in this paper has great potential to guide DMSO applications in vivo.

## 2. Materials and Methods

### 2.1. HER-NIRF System

**Figure 1:**
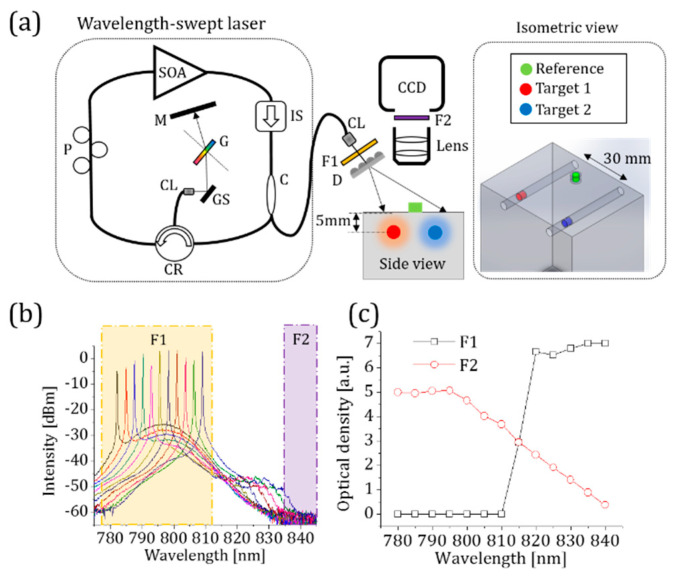
(**a**) A schematic diagram of the wavelength-swept laser and our HER-NIRF system. SOA: semiconductor optical amplifier, P: polarization controller, CR: circulator, CL: collimator, GS: Galvo-scanner, G: grating, M: mirror, C: coupler, IS: isolator, F1: exciter bandpass filter, D: diffuser, F2: emitter bandpass filter, CCD: charge-coupled camera. (**b**) Measured output spectra of our wavelength-swept laser at different wavelengths indicated with different colors. (**c**) A graph of the optical density of F1 and F2 over the wavelength range of interest.

The use of near-infrared wavelength-swept lasers for biomedical imaging has been documented previously [26,27]. We previously developed a new type of wavelength-swept laser with output optimized around 800 nm to excite ICG around its maximum absorption wavelength [28]. It has a holographic transmission grating and a Galvo scanner utilized as a wavelength selector to overcome the thermal instability induced by the traditional piezoelectric-transducer-based wavelength selectors, as shown in Figure 1a. A traveling-wave semiconductor optical amplifier (SOA) is used to give an optical gain in the working spectral range between 784 nm and 805 nm. A 10 dB fiber-optic directional coupler is attached to the SOA’s output port and its 90% arm is utilized for the laser output. The other one, the 10% arm, is linked to the external fiber-optic laser cavity to feedback stimulated light towards the traveling-wave SOA. A holographic transmission grating, a reflection mirror, and a Galvo-scanner mirror comprise the wavelength selector. The output spectrum is measured by an optical spectrum analyzer. The selected spectra of the laser output are displayed in dBm in Figure 1b. The measured −3 dB swept bandwidth of the swept laser is 35 nm (785–820 nm) and the −10 dB swept bandwidth is 40 nm (780–820 nm). The average laser output power per sweep is measured as 4 mW.

The wavelength-swept laser is the core of the HER-NIRF system. A laser-line bandpass filter with a central wavelength of 794 nm, a bandwidth of 32 nm, and an out-of-band optical density (OD) of 7 is employed to clean up non-lasing components. The next component of the HER-NIRF system is an optical diffuser, which homogenizes the Gaussian laser beam with a power of less than 0.5 mW/cm^2^. On the detection side of the HER-NIRF system, a CCD camera (ColdBlue, PerkinElmer optoelectronics, Shelton, CT, USA) with an imaging lens (Marco F/2.8, Sigma Inc., Marlborough, MA, USA) is used to capture snapshots of fluorescence signals. The working distance of the imaging lens is 60 cm. 

To test the HER-NIRF system, an agar phantom mimicking tissue optical properties is used (L 70 mm × W 50 mm × D 100 mm). In the field of biomedical optics, optical phantoms are frequently employed to mimic the optical characteristics of biological tissues [29]. They are created with optical characteristics that are similar to those of living human and animal tissues, such as light scattering and absorption coefficients. Agarose gels can be used to create homogeneous or heterogeneous structures, and their mechanical and physical properties are long-lasting. The turbidity and optical absorption of high-purity agarose gels are relatively low. As a result, the optical properties of agarose-based phantoms might be simply constructed with suitable light scattering and absorption agents [30]. Indian ink is used to adjust its absorption, while Intralipid is added to alter scattering properties. The absorption and reduced scattering coefficients of the phantom are adjusted to 0.0132 mm^−1^ and 1 mm^−1^, respectively. Two holes are prepared 5 mm below the surface of the phantom to be able to insert 3.2 mm (ID) ultra-thin wall glass tubes that are used as inclusions. The tubes are filled with different BSA-ICG and DMSO-ICG solutions.

An emitter bandpass filter that has a central wavelength of 840 nm, a bandwidth of 10 nm, and an average OD of 5 is used to collect the fluorescence emission. Figure 1c shows the measured OD of the filters used in this HER-NIRF system in the absence of ICG. As the wavelength of our swept laser approaches the fluorescence collection band, the performance of our system is degraded by the excitation light leakage. This reduces the signal-to-noise ratio and yields incorrect fluorescence measurements [13].

Therefore, HER-NIRF images need to be corrected by subtracting the excitation-leakage images collected in the absence of ICG. In order to measure the solvatochromic response of ICG, a ratiometric approach is employed to estimate a relative excitation shift in relation to a reference standard that can be either a well-known fluorescent dye or a priori fluoresce image data. By using first-order approximation, the basic equation of fluorescence can be written as

(1) $$F\left(\lambda \right)\approx I\eta \frac{\Omega }{4\pi }2.303\varepsilon (\lambda )lc.$$

where *F* (a.u.) is the steady-state fluorescence intensity, *I* (a.u.) is the incident light excitation intensity on the target, Ω (sr) is the solid angle, *ε* (mol^−1^·cm^−1^) is the molar extinction coefficient as a function of the excitation wavelength *λ* (nm), *η* (%) is the quantum yield, *l* (cm) is the pathlength of excitation light through the fluorescence sample, and c is the concentration of the fluorescent dye.

### 2.2. ICG Characteristics in Water, DMSO, and Bounded to Bovine Serum Albumin

**Figure 2:**
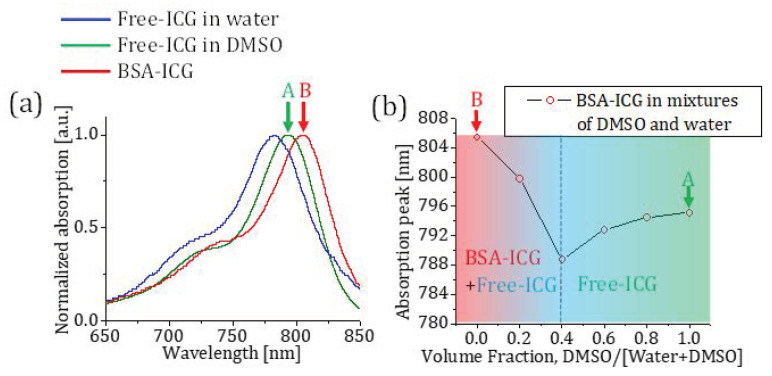
(**a**) The absorption spectra of ICG in different environments. The absorption peaks of BSA-ICG, ICG in DMSO, and ICG in water are 805 nm, 795 nm, and 780 nm, respectively. (**b**) A graph that shows the absorption peak shift of a 6 M BSA-ICG solution when added to different fractions of DMSO and water solutions. The BSA-ICG bond gradually breaks until the volume fraction of 0.4, and after that point, all of ICG becomes free of the BSA-ICG bond.

Typically, ICG is dissolved into an aqueous solution before ICG administration, which has its absorption peak at 780 nm. After the administration, ICG quickly and mostly binds to hydrophobic pockets of albumin, and its absorption peak shifts to 805 nm [9]. We measured the absorption spectra of ICG in different conditions using a commercial spectrometer (USB2000, Ocean Optics Inc., Orlando, FL, USA) in our lab. Figure 2a shows the normalized absorption spectra of ICG in water, DMSO, and bound to bovine serum albumin (BSA-ICG). Figure 1b demonstrates a complex behavior of ICG in mixtures of water, DMSO, and BSA, which mimics ICG in biological media such as lymph or blood in the presence of DMSO. First, ICG is mixed with bovine serum albumin (BSA, Sigma Aldrich, Burlington, MA, USA) with a concentration of 50 mg/mL to mimic the concentration of albumin in human plasma dissolved in distilled water [10]. This mixture is called BSA-ICG. After adding DMSO into the BSA-ICG in distilled water, one can observe that the varying amount of DMSO results in the absorption peak shift of ICG. At the volume fraction zero, the solution contains BSA, ICG, and water. The absorption peak of this solution demonstrates that most of ICG is bound to BSA. 

Our measurements show that the BSA-ICG bond gradually breaks until the volume fraction of 0.4. The solutions in this volume fraction range contain BSA-ICG, free ICG, BSA, water, and DMSO. One can observe that the absorption peak shifts to the shorter wavelength since free ICG starts attributing to this absorption blue-shift. Between the volume fraction of 0.4 and 1, all of the ICG becomes free from the BSA-ICG bond, and its absorption spectrum solely depends on the polarity of the mixtures of solvents.

## 3. Results

### 3.1. The Ratio of Extinction Coefficients of DMSO-ICG and BSA-ICG

The change in the molecular environment affects the wavelength-dependent molecular extinction coefficient of ICG, and the spectral scan driven by our HER-NIRF system reveals the solvatochromic shift. One can assume a simplified case where the change in ICG concentration is trivial between the time sets before and after the administration of DMSO. The ratiometric fluorescence of ICG in DMSO with respect to BSA-ICG in a dilute solution can be written as

(2) $$\ln\left[{F_{D}\left(\lambda \right)/F}_{B}\left(\lambda \right)\right]=\ln\left[\varepsilon_{D}\left(\lambda \right)/\varepsilon_{B}\left(\lambda \right)\right]+\ln\left[\eta_{D}/\eta_{B}\right].\;$$

where the subscripts *B* and *D* describe BSA-ICG and ICG in DMSO, respectively. The right-hand side of Equation (2) consists of a wavelength-dependent ratio of the extinction coefficients and a ratio of the quantum yields.

**Figure 3:**
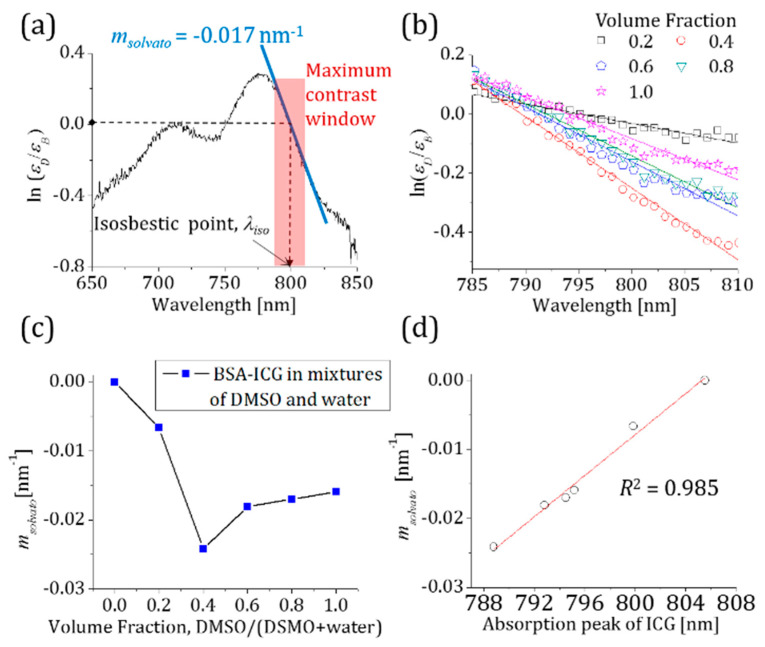
(**a**) The natural logarithmic ratio of the extinction coefficients of DMSO-ICG and BSA-ICG. A blue solid line shows a linear fit to the natural logarithmic ratio obtained with a wavelength-swept laser between 785 nm and 810 nm (red-boxed area). (**b**) A graph shows the slope change of a 6 μM BSA-ICG solution when different amounts of DMSO are added to change the volume fraction. (**c**) The slope values of our linear model for different volume fractions of DSMO. (**d**) The change in the slope values with respect to the ICG absorption peak is nearly perfectly linear.

In order to confirm Equation (2), we utilized the wavelength-swept laser to measure the absorption of DMSO-ICG and BSA-ICG at selected wavelengths in its operational range. The ratio of extinction coefficients of these two ICG solutions is shown in Figure 3a. Previous optical spectrometer absorption spectra measurements performed on the same DMSO-ICG and BSA-ICG solutions are used as the control (Figure 2). Between 785 nm and 810 nm, the natural logarithmic ratio of extinction coefficients shows the maximum contrast and an almost linear decrease as a function of wavelength. In addition, the isosbestic point of DMSO-ICG and BSA-ICG is found in the spectral range. Hence, we can model the ratio of extinction coefficients as a linear function of the excitation wavelength:

(3) $$\ln\left[\varepsilon_{D}\left(\lambda \right)/\varepsilon_{B}\left(\lambda \right)\right]\approx m_{solvato}(\lambda -\lambda_{iso}).$$

where *m_solvato_* (nm^−1^) is the slope of our linear approximation and *λ_iso_* (nm) is the isosbestic wavelength of DMSO-ICG and BSA-ICG in water solutions. In the maximum contrast window, the ratios of extinction coefficients in the different volume fractions are presented in Figure 3b. Calculated values of *m_solvato_* the various volume fractions are plotted as a function of the volume fraction in Figure 3c. One can compare Figure 2b with Figure 3c to find a good correlation between our commercial spectrometer absorption measurements and the slope values of our linear model in Equation (3) shown in Figure 3d. Note that a 1 nm increase in the absorption peak of ICG corresponds to a slope change of 1.45 × 10^−3^ nm^−1^ in our ratiometric model.

### 3.2. Phantom Experiment Results

To conduct tissue-like turbid medium experiments, an Intralipid gelatin phantom is used (μ_a_ = 0.0132 mm^−1^, μ_s_’ = 1 mm^−1^ at 800 nm). Two 3.2 mm long tubes, with a 3.2 mm inner diameter, filled with 26 μL of BSA-ICG and DMSO-ICG solutions are used for the experiments. A third identical BSA-ICG tube is used as the reference. In the first set of experiments, all tubes are placed on top of the phantom for free-space measurements to eliminate the effect of scattering. Following that, the BSA-ICG and DMSO-ICG tubes are placed deep inside the phantom, while the reference BSA-ICG tube is kept on the surface. This arrangement allows us to identify the effect of scattering on the CCD fluorescent measurements at different wavelengths. Despite the shift in the spectrum due to scattering, our method can separate BSA-ICG and DMSO-ICG successfully based on the solvatochromic shift in the ICG fluorescence data.

#### 3.2.1. Free-Space Measurements Using CCD

Prior to phantom experiments, the ratiometric fluorescence analysis is performed in free space using the CCD camera and the very same tubes. These measurements are unaltered and free of the spectral shift in the fluorescence signal due to the migration of photons in a wavelength-dependent high-scattering environment. Hence, these measurements can be used to confirm the previous measurements in Section 3.1, but using the HER-NIRF imaging set-up. 

**Figure 4:**
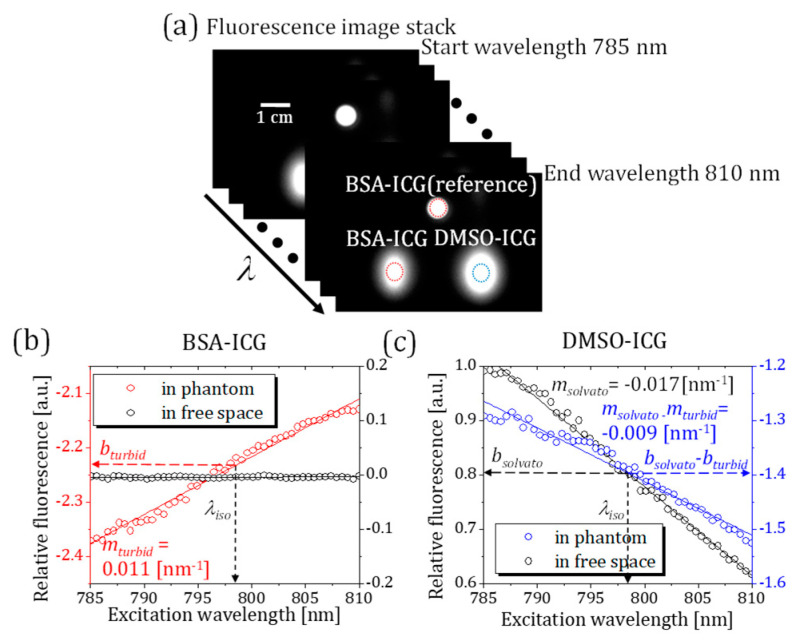
(**a**) The fluorescence image stack consists of 50 images obtained at different excitation wavelengths indicated by the arrow. Red-dashed circles indicate the reference BSA-ICG on the surface and the BSA-ICG embedded in the phantom. The blue-dashed circle shows the DMSO-ICG region of interest. (**b**) The ratiometric measurements of BSA-ICG in free space and phantom with respect to the reference BSA-ICG. (**c**) The ratiometric measurements of DMSO-ICG in free space and phantom with respect to the reference BSA-ICG.

For this purpose, both BSA-ICG and DMSO-ICG tubes are placed on the top surface of the phantom together with the reference BSA-ICG tube, directly under the CCD camera. Fluorescence images at 50 different excitation wavelengths are acquired with a wavelength step of 0.5 nm. The mean fluorescence signals are extracted from regions of interest and used for computation of the ratiometric fluorescence and hence the verification of Equation (2). By incorporating Equation (3) into Equation (2), we obtain

(4) $$\ln\left[\varepsilon_{D}\left(\lambda \right)/\varepsilon_{B}\left(\lambda \right)\right]+\ln\left[\eta_{D}/\eta_{B}\right]=m_{solvato}\left(\lambda -\lambda_{iso}\right)+b_{quantum}.$$

where *b_quantum_* is the wavelength-independent value of the relative quantum yield ratio. As seen from the black dots and line in Figure 4c, *b_quantum_* can be read at the isosbestic point and is 0.80, which means $\eta_{D}$ is 2.23 times higher than $\eta_{B}$. The calculated *m_solvato_* obtained from the relative fluorescence measurement in Figure 4c is −0.017 nm^−1^, which shows good agreement with the calculated *m_solvato_* from our absorption measurements without imaging set-up, as shown in Figure 3a. As expected, the slope value between two identical fluorescence signals of the BSA-ICG tube and the reference BSA-ICG tube yields approximately zero, as shown by the black dots and line in Figure 4b. 

#### 3.2.2. Phantom Measurements Using HER-NIRF System

The turbid phantom experiment is designed to demonstrate the DMSO-ICG sensing performance of our HER-NIRF technique with spectral distortion by multiple scattering. While the reference BSA-ICG tube is kept on top of the phantom, the second BSA-ICG tube is placed in the turbid phantom at a 5 mm depth as an experimental control (Figure 1a). The other tube filled with DMSO-ICG is also placed in the phantom at the same depth of the embedded BSA-ICG tube but 30 mm apart. Again, fluorescence images at 50 different excitation wavelengths are acquired with a wavelength step of 0.5 nm. The CCD camera integration time is 1 s per image. 

Since the tubes are placed deep inside the turbid media, the spot sizes detected at the top surface of the phantom are much larger due to scattering (Figure 4a). The mean fluorescence signals are measured at the selected regions of interest indicated by the dashed-line circles in Figure 4a. The ratiometric fluorescence for BSA-ICG and DMSO-ICG tubes with respect to the BSA-ICG reference is plotted as a function of the excitation wavelength as red and blue dots and lines in Figure 4b,c, respectively. The ratiometric fluorescence between the reference BSA-ICG on the surface and the BSA-ICG embedded in the phantom demonstrates a red-shift due to the multiple scattering, which yields a positive slope value in our ratiometric model, as shown in Figure 4b. Likewise, the multiple scattering shifts the measured relative excitation spectrum of DMSO-ICG by changing the slope value from −0.017 nm^−1^ (in free space) to −0.009 nm^−1^ (in phantom).

#### 3.2.3. HER-NIRF Spectral Map for DSMO Sensing

A fluorescence signal from an embedded ICG tube in a turbid medium can be written as

(5) $$A\left(F\right)=F\exp\left[-\mu_{eff}\left(\lambda \right)2d\right].$$

where *A* is the attenuation operator describing Beer’s law, *μ_eff_* is the wavelength-dependent effective attenuation coefficient (mm^−1^), and *d* (mm) is the depth of the tube. The factor two describes the photon round trip. If both the reference and the embedded targets contain the same BSA-ICG, we obtain

(6) $$\ln\left[\frac{A\left(F_{B}\right)}{F_{B}}\right]=-\mu_{eff}\left(\lambda \right)2d=m_{turbid}\left(\lambda -\lambda_{iso}\right)+b_{turbid}.$$

where *m_turbid_* is the slope of the linear fit in Figure 4b that is equal to 0.011 nm^−1^. The parameter *b_turbid_* is −2.23, which describes the wavelength-independent attenuation in the region of interest. If the embedded tube contains a different solution (DMSO-ICG) than the reference (BSA-ICG), we can use Equations (2)–(6) to obtain

(7) $$\ln\left[\frac{A\left(F_{D}\right)}{F_{B}}\right]=\left(m_{solvato}-m_{turbid}\right)\left(\lambda -\lambda_{iso}\right)+(b_{quantum}-b_{turbid}).$$

In Figure 4c, $m_{solvato}$ corresponds to the slope of the black dots and line, while $\left(m_{solvato}-m_{turbid}\right)$ corresponds to the slope of the blue dots and line. The spectral distortion by multiple scattering affects the spectral content of DMSO-ICG measurements, similar to the result shown in Figure 4b. 

**Figure 5:**
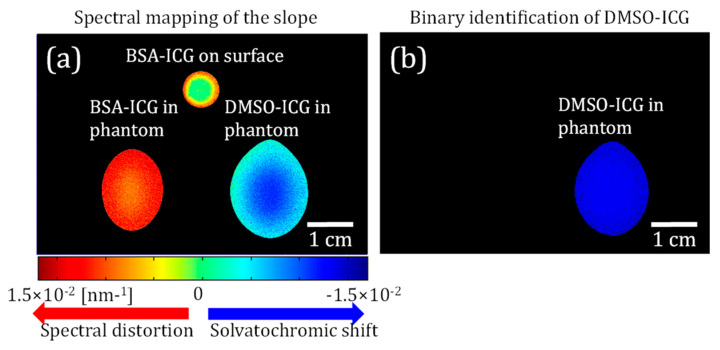
(**a**) A color-coded slope map shows the excitation blue-shift of the DMSO-ICG tube while the embedded BSA-ICG sample demonstrated a red-shift of the excitation spectrum by multiple scattering. Low-signal pixels from the intercept map are excluded from the image processing. (**b**) A binary identification map exclusively displays DMSO-ICG. All the pixels with slope values that are lower than a slope threshold are removed from the original spectral map.

Based on this theory and our experimental results, we further applied our ratiometric technique to produce a pixel-by-pixel spectroscopic map. First, the fluorescence signal at each pixel is used as a numerator on the left-hand side of Equation (7), while the fluorescence signal of the reference BSA-ICG on the surface is used as the denominator. Afterward, the slope of the linear fit per pixel is attributed to the pixel under study to produce a spectroscopic image, as shown in Figure 5a. When the pixels show a slope of approximately zero, they identify fluorescence signals from the reference BSA-ICG on the surface. However, when the pixels show a positive slope, they belong to the multiple scattered fluorescence signals from the embedded BSA-ICG tubes. 

Note that a positive slope is observed even around the container wall of the reference BSA-ICG due to scattering within the contained wall. A slope threshold of our linear fitting (−2.95 × 10^−5^ nm^−1^) is applied on the spectral map to generate a binary identification map of DMSO-ICG. Despite this spectral content degradation induced by multiple scattering, one can still identify the pixels of DMSO-ICG tubes in Figure 5b. These successful results show that the HER-NIRF system is a great tool for mapping DSMO distribution in thick turbid media.

## 4. Discussion

In this work, we successfully demonstrate the potential of our proof-of-concept HER-NIRF system in monitoring DMSO distribution in turbid media. The spectroscopic measurement of the local polarity expands the information contents of conventional wide-field fluorescence imaging techniques. Our method is based on the solvatochromic shift of ICG, which depends on the local environment. The DMSO amount in the local area regulates the amount of the solvatochromic shift, and the HER-NIRF system measures the extent of this shift to determine the DSMO amount in return. This methodology works perfectly in free space; however, the effect of scattering on the measured signals should be considered in a turbid medium such as biological tissue. For this purpose, free-space measurements are performed first prior to using tissue-simulating agar phantom, which is designed to mimic in vivo conditions. Different ICG solutions are embedded deep in the phantom to demonstrate the effect of the scattering on the measured fluorescence spectrum. Since the scattering in tissue is dominated by mie-scattering, it is wavelength-dependent [31]. Hence, the lower wavelengths experience higher scattering that creates a red-shift in the fluorescence signals traveling from the ICG target to the surface. 

Since the HER-NIRF system differentiates the DSMO solution by the solvatochromic shift that resembles a blue-shift, scattering distorts the spectral information in the opposite way. Regardless, our measurements demonstrate that our HER-NIRF system can differentiate DSMO-ICG solution. Although the sign of the slope is used to qualitatively identify the presence of DMSO in each pixel, our measurements show that quantitative imaging can be possible if one can choose appropriate reference signals to minimize spectral distortion. However, a reference point at the same depth may not be available for all applications. In such cases, one alternative strategy might be performing HER-NIRF imaging before and after the application of DMSO. Hence, determination can be made by considering the change in the slope of the ratiometric fluorescence of the same target. For example, if ICG is accumulated in lymph nodes and imaging is performed before and after the application of DMSO, the technique presented here can be applied since the depth of the reference and target will be the same. 

The wavelength-swept laser is the key for our HER-NIRF technique due to its superior performance compared to other available light sources [16]. Being low-cost, fast in the spectral scan, and able to perform coherent light amplification, this wavelength-swept laser can be a very powerful light source for real-time measurement in various DSMO sensing/mapping applications. While its superior spectral resolution is a major advantage for this application, the HER-NIRF technique also provides a much higher signal-to-noise ratio by measuring the whole fluorescence emission spectrum as opposed to conventional systems utilizing multiple or variable bandpass filters at the detection site. This will be important, particularly for the tomographic HER-NIRF application, where speed is important [25,32,33,34]. For in vivo applications that require higher power, optical amplifiers can be utilized to amplify the signal output of the wavelength-swept source. Future tomographic applications will also allow 3D imaging as opposed to 2D mapping of DMSO demonstrated in this study.

One of the strengths of our technique is that the wavelength-swept laser at the core of our system works in the NIR range, centered around 800 nm, where auto-fluorescence from the tissue components diminishes. Hence, our technique is immune to the contamination of the auto-fluorescence signals originating from various tissue components. On the other hand, there are some weaknesses in our system that should be noted. As seen in Figure 1c, the emitter bandpass filter F2 has a significantly larger bandwidth than its originally stated bandwidth of 10 nm. This is due to the fact that the performance of the fluorescence emission filters unfortunately depends on the incident angle of the incoming photons. The performance of these filters is optimum when the photons hit the filter perpendicularly. However, in our camera-based system, they are positioned between the lens and the CCD camera, which makes their performance far from ideal. While the lens is focusing the image on to the CCD camera, different portions of the light beam pass through the filter at various angles, which deteriorates the performance of the filter. Although there might be some ways to collimate the beam while passing through an emission filter, in practical imaging applications, this is the typical configuration, so we chose to test the system under these imperfect conditions [17,35,36,37].

It is important to evaluate the detection limits of this technique. The error in the measured slopes, m_solvato_ and m_turbid_, is around 11% (0.0018 nm^−1^). This error is nearly five times smaller than the change in the slope due to the blue-shift from the solvatochromic shift in DSMO, despite the opposite red-shift in the fluorescence signals traveling from the ICG target to the surface, m_solvato_ − m_turbid_ (0.0090 nm^−1^). Therefore, the method of applying thresholding worked, as shown in our study. It should be noted that the red-shift due to the scattering depends on the depth of the inclusions and the scattering of the medium. Therefore, the signal generated by the deeper inclusions will experience a higher red-shift and at some point, when the error in the measured slopes is comparable to the m_solvato_ − m_turbid_, our technique will reach its detection limit. Since there are two parameters that govern this limit, the reduced scattering coefficient of the medium and the depth of the inclusion, the depth limit of our technique will depend on the tissue type. However, it might be safe to state that in regular tissues, our technique will be limited to 1 cm. 

The ability to measure the distribution of DMSO in vivo can lead to a better understanding of the optimum DSMO concentration required for many therapeutic and drug delivery applications. Furthermore, such a system can guide those applications in real time by optimizing the DSMO dose in the future. On the other hand, this technique can also help increase the knowledge of the pharmacokinetic properties of ICG in tissue. For instance, K. Licha et al. demonstrated that increasing the hydrophilicity of cyanine dyes enhances the fluorescence image contrast of tumors by inhibiting the plasma protein binding of these dyes [14]. Thus, the properties of DMSO inhibiting the albumin binding of ICG can lead to a better understanding of the pharmacokinetics of ICG for tumor imaging. Finally, by providing additional spectral information, the HER-NIRF technique can be applied for measuring the solvatochromic response of other fluorescence dyes and even be adapted for endoscopic applications due to efficient light guiding by fiber optics. 

## 5. Conclusions

In this study, we demonstrate a steady-state spectroscopic wide-field imaging technique that has the ability to resolve the solvatochromic response of ICG. The pivotal part of our imaging technique is a continuous-wave near-infrared novel wavelength-swept laser, which can tune its output wavelength with high speed and superior spectral resolution. The solvatochromic response of ICG depends on the local DMSO concentration. Hence, this HER-NIRF system can map the DMSO concentration in biological tissues and will be crucial in determining the best DMSO dose for a specific application to improve medication delivery effectiveness and minimize adverse responses. More importantly, this technology may also direct the DMSO dose in real time during these therapeutic applications. 

## Data Availability

The data presented in this study are available on request from the corresponding author.
